# NOTCH2NLC-related oculopharyngodistal myopathy type 3 complicated with focal segmental glomerular sclerosis: a case report

**DOI:** 10.1186/s12883-022-02766-3

**Published:** 2022-07-04

**Authors:** Guang Ji, Yuan Zhao, Jian Zhang, Hui Dong, Hongran Wu, Xian Chen, Xiaoming Qi, Yun Tian, Lu Shen, Guofeng Yang, Xueqin Song

**Affiliations:** 1 Department of Neurology, The Second Hospital of Hebei Medical University, Shijiazhuang, Hebei China; 2 Department of Geriatrics, The Second Hospital of Hebei Medical University, Shijiazhuang, Hebei China; 3 Department of Nephropathy, The Second Hospital of Hebei Medical University, Shijiazhuang, Hebei China; 4 Department of Neurology, Xiangya Hospital, Central South University, Changsha, 410008 Hunan China

**Keywords:** Oculopharyngodistal myopathy (OPDM), NOTCH2NLC, Eosinophilic intranuclear inclusions, Focal segmental glomerular sclerosis (FSGS), GGC repeat expansion

## Abstract

**Background:**

Oculopharyngodistal myopathy (OPDM) is an adult-onset neuromuscular disease characterized by progressive ocular, facial, pharyngeal, and distal limb muscle involvement. Recent research showed that GGC repeat expansions in the NOTCH2NLC gene were observed in a proportion of OPDM patients, and these patients were designated as having OPDM type 3 (OPDM3). Heterogeneous neuromuscular manifestations have been described previously in studies of OPDM3; however, kidney involvement in this disease has rarely been reported.

**Case presentation:**

Here, we report the case of a 22-year-old Chinese patient with typical manifestations of OPDM complicated with focal segmental glomerular sclerosis (FSGS). This patient with sporadic FSGS exhibited distal motor neuropathy and rimmed vacuolar myopathy in clinical and pathological examinations. An expansion of 122 CGG repeats located in the 5’ untranslated region (UTR) of the NOTCH2NLC gene was identified as the causative mutation in this patient. The clinical and histopathological findings fully met the criteria for the diagnosis of OPDM3. In addition, intranuclear inclusions were detected in the renal tubule epithelial cells of this patient, indicating that the kidney may also be impaired in NOTCH2NLC-related GGC repeat expansion disorders (NREDs).

**Conclusions:**

Our case report demonstrated the clinicopathological cooccurrence of sporadic FSGS and OPDM3 in a patient, which highlighted that the kidney may show inclusion depositions in OPDM3, thus expanding the clinical spectrum of NREDs.

## Background

Oculopharyngodistal myopathy (OPDM) is a rare, clinicopathologically distinct muscular disease. The typical clinical manifestations are insidiously progressive ptosis, ophthalmoparesis, facial and masseter weakness, dysphagia, and distal limb muscle weakness [1]. Myopathological findings include rimmed vacuoles and chronic myopathic changes without myonecrosis or inflammation [2]. Since first described in 1977 [3], OPDM has affected more than 300 individuals worldwide [1, 2, 4–9]. To date, three causative genes have been identified for OPDM, LDL receptor-related protein 12 (LRP12), GAIP/RGS19-interacting protein (GIPC1), and NOTCH2NLC, which are responsible for OPDM1, OPDM2, and OPDM3, respectively [9–12]. The diagnosis of OPDM previously depended on clinical manifestations, histopathological findings, and genetic exclusion of similar conditions. Ultrastructural examinations of the central and peripheral nervous system tissues, skeletal muscles, and skin show abundant round, eosinophilic intranuclear inclusions (positive for ubiquitin and p62) [13, 14]. Here, we report the case of a 22-year-old man with progressive aggravating limb weakness and moderately increased creatine kinase in serum. GGC repeat expansions in the 5’-UTR of the NOTCH2NLC gene further confirmed the diagnosis of OPDM3. Interestingly, we observed intranuclear inclusions in the renal tubule epithelial cells of this patient, which indicated that the kidney, in addition to the central and peripheral nervous systems, may be involved in NOTCH2NLC-related OPDM3.

## Case presentation

A 22-year-old patient presenting with progressive aggravating limb weakness for more than 7 years was hospitalized because of a high creatine kinase (CK) level in serum. During the 7 years before admission, the patient exhibited gradual deterioration of limb strength, as well as slurred speech and dysarthria. At first, he had trouble squatting and was prone to ankle sprains. Four years later, he began to walk unsteadily, fall easily, and have difficulty climbing stairs. His handwriting also became clumsy. After one year, he complained of swallowing difficulty, especially when taking solid food. The parents brought the patient to our hospital, as his clinical symptoms showed a tendency of progressive worsening. In addition, one year before being admitted to our unit, the patient had been hospitalized for proteinuria and was diagnosed with FSGS based on the findings of a renal biopsy sample evaluation, and he received oral benazepril 5 mg daily after discharge. His mother stated that the parents did not exhibit any similar symptoms, while one of his uncles was born with mental retardation.

On admission to our unit, we did not detect any problems in a general physical examination. The patient displayed facial weakness, mild palpebral ptosis, and limited abduction of the right eye, with dysarthria and slurred speech. His gag reflex was also weakened. According to the Medical Research Council (MRC) score, the proximal muscle strength in his upper and lower limbs was 5/5. The MRC score in the distal upper limbs was 4^+^/5, while it was 4/5 in the distal lower muscles. His muscle tone was normal, but the tendon reflexes were absent. The young man showed bilateral gastrocnemius atrophy, as well as interosseus, thenar, and hypothenar muscle atrophy. Babinski signs were noted bilaterally. Sensation and coordination were intact. No severe cognitive impairments were identified in this patient. He scored 27/30 on the Mini-Mental State Examination (MMSE) and scored 27/30 on the Montreal Cognitive Assessment (MoCA). Routine blood tests revealed that the patient's erythrocyte count was low at 4.22X10^12^/L (normal, 4.3–5.8 × 10^9^/L), and the haemoglobin level was 122 g/L (normal, 130–175 g/L). A twenty-four hour urinary protein quantification showed an increased protein level of 3.22 g/d. Laboratory investigations also revealed an elevated serum CK level of 1505.9 U/L (normal 50–310 U/L). The results of the other laboratory tests were unremarkable.

**Fig. 1:**
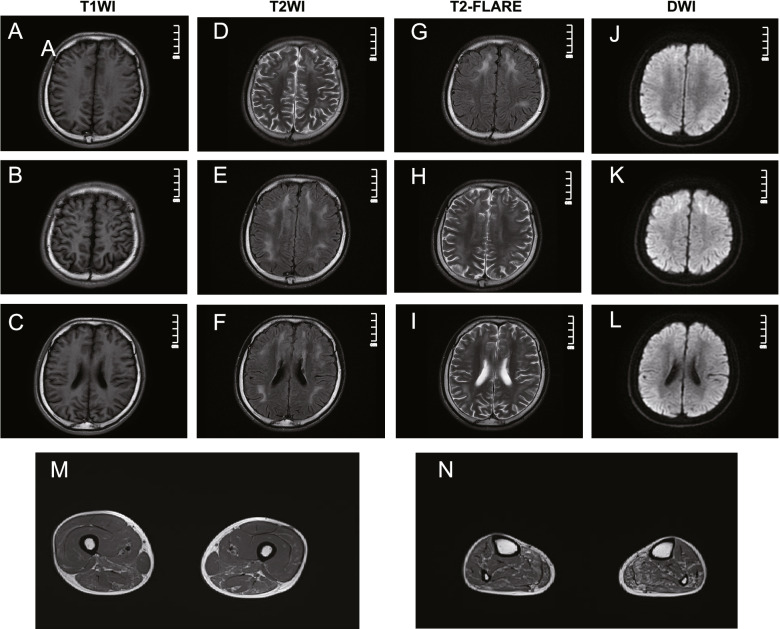
Brain (**A-L**) and muscle MRI (**M**, **N**) findings. Brain MRI revealed bilateral subcortical high-intensity lesions in the centrum semiovale and anterior and posterior horns of the lateral ventricle on T2WI (**D**, **E**, **F**) and FLAlR (**G**, **H**, **I**) images. The corresponding lesions were characterized by high signal intensity on DWI sequences (**J**, **K**, **L**). Muscle MRI showed fatty infiltration and the atrophy of the lower limb muscles. The distal muscles (**N** calf level) were more severely affected than the proximal muscles (**M** thigh level), and the posterior muscles were more severely affected than the anterior muscles

Brain magnetic resonance imaging (MRI) revealed diffuse leukoencephalopathy on T2-weighted fluid-attenuated inversion recovery (T2-FLAIR) and high-intensity signals along the corticomedullary junction on diffusion-weighted imaging (DWI), while no abnormal findings on apparent diffusion coefficient (ADC) mapping were observed (Fig. 1A-L). Muscle MRI of the patient showed fatty infiltration and muscle atrophy of the lower limb muscles (Fig. 1M, N). Needle electromyography showed a long duration, high amplitude, and decreased phase number of motor unit action potentials (MUAPs), which indicated neurogenic changes in the tested muscles. Nerve conduction studies (NCSs) revealed reduced conduction velocities in all tested motor and sensory nerves and prolonged distal latencies in the right middle median and ulnar nerves. Decreased compound muscle action potentials were observed in the left peroneal nerve and bilateral tibial nerves.

**Fig. 2:**
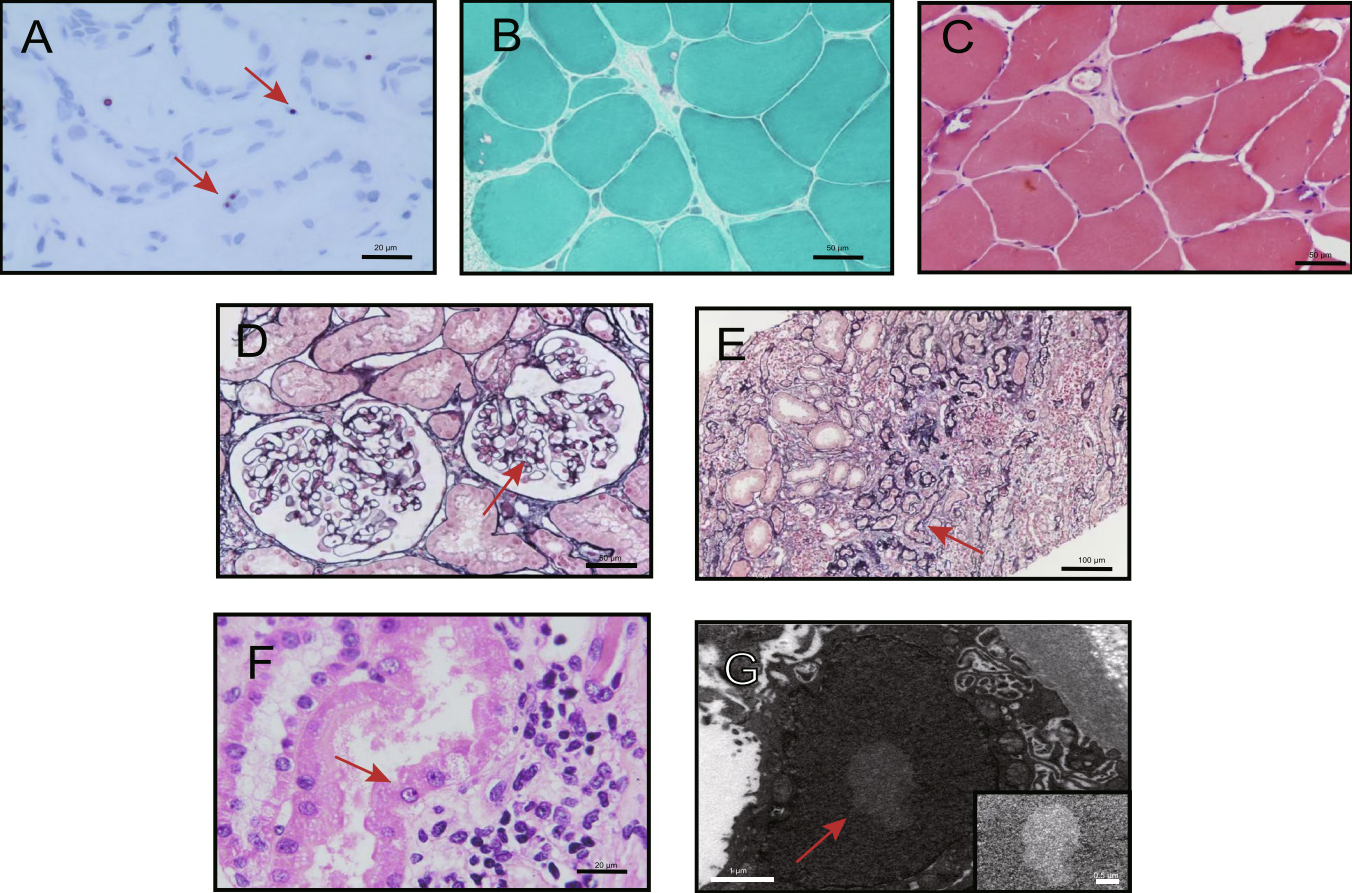
Pathological changes in the skin, muscle, and kidney biopsy samples. Cells were imaged with an Olympus BX51 microscope with a DP72 camera and the Plan achromat objective, and images were acquired by CellSens software. For electron microscopy, cells were observed with a transmission electron microscope (TEM, JEM 1230, JEOL) with a CCD electronic imaging system, and images were acquired by Gatan Digital Micrograph software. **A** Immunohistochemical staining showed that the inclusion bodies in the skin biopsy sample were positive for p62 (bar = 20 μm, 1000 ×). **B**, **C** Rimmed vacuoles were present in the muscle fibres of mGT and H&E stained Sects. (400 × bar = 50 μm). **D**, **E** Renal pathological characteristics in PASM plus Masson stained sections (In **D**, bar = 50 μm, 400 × ; In **E**, bar = 100 μm, 200 ×). The arrow in (**D**) shows segmental sclerosis. The arrow in (**E**) shows the localized tubular atrophy. **F** H&E staining of renal tubular epithelial cells (bar = 20 μm, 1000 ×). The arrow indicates eosinophilic inclusion bodies. **G** Electron microscopy of renal tissues demonstrated intranuclear inclusions without membranes (bar = 1 μm, 10 K ×) (marked by arrow and shown at higher magnification)

The evaluation of the skin biopsy sample revealed eosinophilic, p62-positive, intranuclear inclusions in adipocytes, sweat gland cells, and fibroblasts (Fig. 2A). The evaluation of the quadriceps biopsy sample revealed rimmed vacuolar fibres and a small proportion of angular atrophic fibres, but no hypertrophic fibres, necrosis, inflammatory infiltration, or ragged red fibres, which would suggest mitochondrial dysfunction, were observed (Fig. 2B, C). A renal biopsy was performed, and an evaluation of the biopsy sample revealed segmental sclerosis and localized tubular atrophy (Fig. 2D, E). Periodic acid silver methenamine (PASM) plus Masson trichrome (MASSON) staining showed that of the 12 identified glomeruli, four were found to have segmental sclerosis, and four had global sclerosis. Cellularity was absent, and membrane thickness was normal. One-third of renal tubules were atrophied, with corresponding renal interstitial fibrosis and lymphomonocyte infiltration. Haematoxylin and eosin (H&E) staining showed eosinophilic intranuclear inclusions in renal tubular epithelial cells (Fig. 2F,). Electron microscopy further clarified inclusions without limiting membranes in renal tubular epithelial cells (Fig. 2G).

**Fig. 3:**
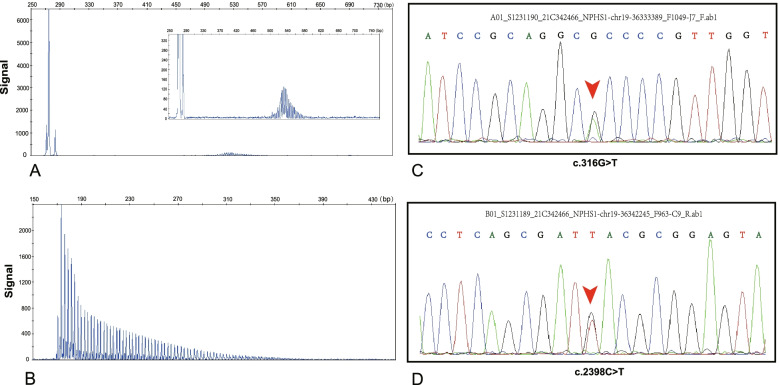
**A**, **B** Validation of GGC repeat expansions in the NOTCH2NLC gene by GC-PCR (**A**) and RP-PCR (**B**). **C**, **D** Sanger sequencing of the patient. Two heterozygous mutations, c.316G > T (p.D106Y) and c.2398C > T (p.R800C), in the NPHS1 gene were recognized by genetic testing. The mutation c.316G > T was identified within exon 3 of NPHS1 (**C**). The mutation c.2398C > T was identified within exon 18 of NPHS1 (**D**)

Genetic analysis using repeat-primed polymerase chain reaction (RP-PCR) and polymerase chain reaction amplification for GC-rich regions (GC-PCR) were performed to confirm the diagnosis [15]. Genetic analysis revealed 121 CGG expansions in the 5’-untranslated region of NOTCH2NLC (Fig. 3A, B). In addition, the coding exons of 804 genes associated with diseases of the urinary system were selected by a gene capture strategy using the GenCap custom enrichment kit (MyGenostics, Beijing), and enriched libraries were sequenced on an Illumina NextSeq 500 sequencer (Illumina, San Diego, CA, USA) for paired-end reads of 150 bp. Two missense mutations (c.316G > T in exon 3 and c.2398C > T in exon 18) were identified in the NPHS1 gene, including one mutation that had been reported in the Human Gene Mutation Database (HGMD) to be associated with nephrotic syndrome. The mutational sites were confirmed by Sanger sequencing (Fig. 3C, D), but verification by Sanger sequencing was not performed for his parents. At the one-year follow-up after discharge, the patient did not re-examine his proteinnuria, and his muscle strength had progressively reduced.

## Discussion and conclusions

Oculopharyngodistal myopathy is considered to be a rare, adult-onset hereditary muscle disease with both autosomal-dominant and autosomal-recessive inheritance patterns. Three causative genes have been identified for OPDM. According to the genetic defect, this disease has been categorized into OPDM1, OPDM2, and OPDM3, which are associated with 5’-UTR CGG repeat expansions in LRP12, GIPC1, and NOTCH2NLC, respectively [9–11]. This patient exhibited the typical clinical manifestations of OPDM, including dysarthria, dysphagia, facial weakness, ptosis, and weakness in distal limbs. Rimmed vacuoles in H&E and mGT stained sections indicated that the dominant myopathic changes of OPDM were present in this patient. Gene analysis identified CGG expansions in the 5’-untranslated region of NOTCH2NLC, which further confirmed the diagnosis of OPDM3. In addition, the patient displayed peripheral neuropathy in electromyography and hyperintense linear lesions in corticomedullary junctions in DWI, which are thought to be typical changes of adult-onset NIID [16]. However, this patient did not exhibit typical syndromes of adult-onset NIID, such as dementia, episodic encephalopathy, or parkinsonism. Yu et al. reported the presence of peripheral neuropathy and white matter changes in a proportion of OPDM3 patients and proposed that subclinical white matter involvement may be due to the long disease duration [9].

The expansion of CGG repeats in NOTCH2NLC was first identified as the genetic cause of neuronal intranuclear inclusion disease (NIID)[11, 17, 18]. Subsequently, the pleiotropy of NOTCH2NLC was identified as being causative for many types of neurodegenerative diseases [15, 19, 20]. Recent research further expanded the clinical spectrum of NOTCH2NLC-related disorders to OPDM3[9] and other peripheral neuropathies and myopathies [13, 21]. An increasing number of studies have shown evidence of NOTCH2NLC CGG repeat expansions in various central and peripheral nervous system disorders; however, descriptions of systemic organ disorders have rarely been recognized in previous reports.

In addition to the manifestations of OPDM, our patient presented with proteinuria, and typical pathognomonic characteristics of FSGS were observed in the patients’ renal biopsy samples. Interestingly, eosinophilic intranuclear inclusions were found in renal tubular epithelial cells. We wondered whether there was a linkage between FSGS and GGC repeat expansion in NOTCH2NLC or whether this finding was just a coincidence. Next-generation sequencing identified two mutations in NPHS1, which is a commonly mutated gene in patients with sporadic FSGS [22]. However, whether the identified genetic variants actually contribute to disease remains to be further validated. It was also noted that this patient developed OPDM at an earlier age than in the published reports of recent case studies, and whether the mutations in NPHS1 exerted an accelerating effect on the onset of OPDM remains unclear. Previously, intranuclear inclusions had been reported in renal tubular cells in a renal biopsy sample obtained 12 years preceding the diagnosis of NIID, and the patient presented with mesangioproliferative glomerulonephritis [23]. A case of an NIID patient who showed lupus nephritis–like pathology in renal biopsy sample in whom proteinuria improved after proper treatment has also been reported [24]. However, information on NOTCH2NLC CGG repeat expansions was not acquired in these reports. Interestingly, a general autopsy of an OPDM1 patient with CGG repeat expansions in LRP12 revealed that almost all organs, including the kidney, showed abundant round, eosinophilic intranuclear inclusions, suggesting that the disease process could affect various extramuscular organs, but further studies are required to clarify whether the extramuscular organ lesions induced associated symptoms [14].

Recently, in view of the coexistent GGC repeat expansions in NOTCH2NLC and the above phenotypes, the term NOTCH2NLC-related GGC repeat expansion disorders (NREDs) was used to summarize all diseases caused by the GGC repeat expansions of NOTCH2NLC, regardless of their diverse clinical manifestations [25]. As described by Boivin et al. [12], NOTCH2NLC GGC repeats are mainly translated into a polyG-containing protein called uN2CpolyG (upstream of N2C polyG-containing protein). The uN2CpolyG proteins are responsible for intranuclear inclusions and are toxic in cells and tissue samples. These novel genetic disorders caused by NOTCH2NLC GGC expansion are named polyG diseases. However, the underlying mechanisms of cell death caused by uN2CpolyG proteins are unclear. Since NOTCH2NLC is a human-specific gene, establishing appropriate models to study NREDs is still a major challenge [15].

In summary, we report the case of an OPDM3 patient complicated with FSGS in whom we confirmed the presence of intranuclear inclusions in kidney tissues, supporting the possibility that NREDs are spectrum disorders involving widespread systemic organs.

## Data Availability

The datasets for this article are not publicly available due to concerns regarding participant/patient anonymity. Requests to access the datasets should be directed to the corresponding author.

## Abbreviations

OPDM: Oculopharyngodistal myopathy
FSGS: Focal segmental glomerular sclerosis
NIID: Neuronal intranuclear inclusion disease
NRED: NOTCH2NLC-related GGC repeat expansion disorders
UTR: Untranslated region
LRP12: LDL receptor-related protein 12
GIPC1: GAIP/RGS19-interacting protein
DWI: Diffusion-weighted imaging
MRI: Magnetic resonance imaging
CK: Creatine kinase
MMSE: Mini-Mental State Examination
NCS: Nerve conduction study
PASM + MASSON: Periodic acid silver methnamine + Masson trichrome
GC-PCR: Polymerase chain reaction amplification for GC-rich regions
RP-PCR: Repeat-primed polymerase chain reaction
