# Correction: NOTCH2NLC-related oculopharyngodistal myopathy type 3 complicated with focal segmental glomerular sclerosis: a case report

**DOI:** 10.1186/s12883-022-02810-2

**Published:** 2022-08-08

**Authors:** Guang Ji, Yuan Zhao, Jian Zhang, Hui Dong, Hongran Wu, Xian Chen, Xiaoming Qi, Yun Tian, Lu Shen, Guofeng Yang, Xueqin Song

**Affiliations:** 1 Department of Neurology, The Second Hospital of Hebei Medical University, Shijiazhuang, Hebei China; 2 Department of Geriatrics, The Second Hospital of Hebei Medical University, Shijiazhuang, Hebei China; 3 Department of Nephropathy, The Second Hospital of Hebei Medical University, Shijiazhuang, Hebei China; 4 Department of Neurology, Xiangya Hospital, Central South University, Changsha, 410008 Hunan China

**Correction: BMC Neurol 22, 243 (2022)**

**https://doi.org/10.1186/s12883-022-02766-3**

Following publication of the original article [1], the authors identified an error in Fig. 1, wherein the position of images E,F and images H,I are reversed. The correct figure is given below.

**Fig. 1:**
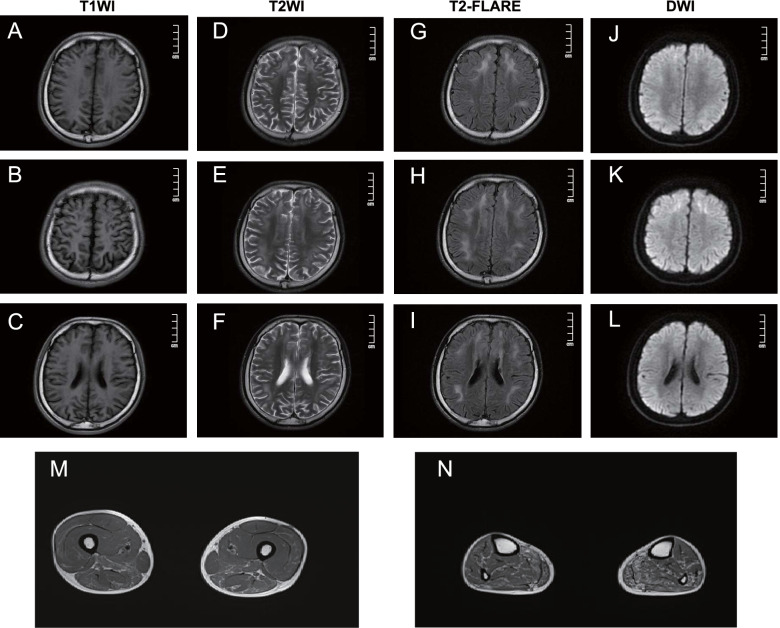
Brain (**A-L**) and muscle MRI (**M, N**) findings. Brain MRI revealed bilateral subcortical high-intensity lesions in the centrum semiovale and anterior and posterior horns of the lateral ventricle on T2WI (**D, E, F**) and FLAlR (**G, H, I**) images. The corresponding lesions were characterized by high signal intensity on DWI sequences (**J, K, L**). Muscle MRI showed fatty infiltration and the atrophy of the lower limb muscles. The distal muscles (**N** calf level) were more severely affected than the proximal muscles (**M** thigh level), and the posterior muscles were more severely affected than the anterior muscles

The original article [1] has been updated.
